# C_4_ photosynthesis: 50 years of discovery and innovation

**DOI:** 10.1093/jxb/erw491

**Published:** 2017-01-21

**Authors:** Susanne von Caemmerer, Oula Ghannoum, Robert T. Furbank

**Affiliations:** 1 Australian Research Council Centre of Excellence for Translational Photosynthesis, Division of Plant Sciences, Research School of Biology, The Australian National University, Acton, ACT 2601, Australia; 2 ARC Centre of Excellence for Translational Photosynthesis and Hawkesbury Institute for the Environment, Western Sydney University, Richmond NSW 2753, Australia

**Keywords:** ^14^C pulse chase, C_3_–C_4_ intermediate species, C_4_ crops, C_4_ photosynthetic pathway, C_4_ rice project, CO_2_ fixation, convergent evolution, Hal Hatch and Roger Slack, Kranz anatomy.

**It is now over half a century since the biochemical characterization of the C_4_ photosynthetic pathway, and this special issue highlights the sheer breadth of current knowledge. New genomic and transcriptomic information shows that multi-level regulation of gene expression is required for the pathway to function, yet we know it to be one of the most dynamic examples of convergent evolution. Now, a focus on the molecular transition from C_3_–C_4_ intermediates, together with improved mathematical models, experimental tools and transformation systems, holds great promise for improving C_4_ photosynthesis in crops.**

The year 2016 marked 50 years since the first published biochemical characterization of the C_4_ photosynthetic pathway by Hal Hatch and Roger Slack (Box 1; Hatch and Slack, 1966). Following the experimental designs of Calvin and co-workers, they used ^14^CO_2_ to trace the fate of CO_2_ assimilated by sugarcane and confirmed that the first carbon compound formed was a C_4_ acid. This led to the definition of the C_4_ dicarboxylic acid pathway, later abbreviated to C_4_ photosynthesis, and the plants employing this process were termed C_4_ plants. Furbank, in one of two comprehensive Darwin reviews in this issue, retraces these historical events in detail (Furbank, 2016). After the seminal experiments by Hatch and Slack, unravelling the biochemistry of the pathway in a number of species followed rapidly and provided the foundation of our current knowledge on the diverse biochemistry of C_4_ photosynthesis (Hatch, 1987; Hatch, 1992; Furbank, 2016).

**Box 1. Pioneers of C_4_ photosynthesis**

Roger Slack, Hilary Warren and Hal Hatch at the opening of the conference ‘C_4_ Photosynthesis: past, present and future’ in April 2016. The meeting was held at the ARC Centre of Excellence for Translational Photosynthesis in Canberra, Australia to commemorate the discovery of the C_4_ pathway and its significance in today’s plant biology and agricultural research (see Hibberd and Furbank, 2016). Hilary Warren (then Johnson) was the first PhD student of Hal Hatch. It is with sadness that we note that Roger Slack passed away on 24 October 2016.

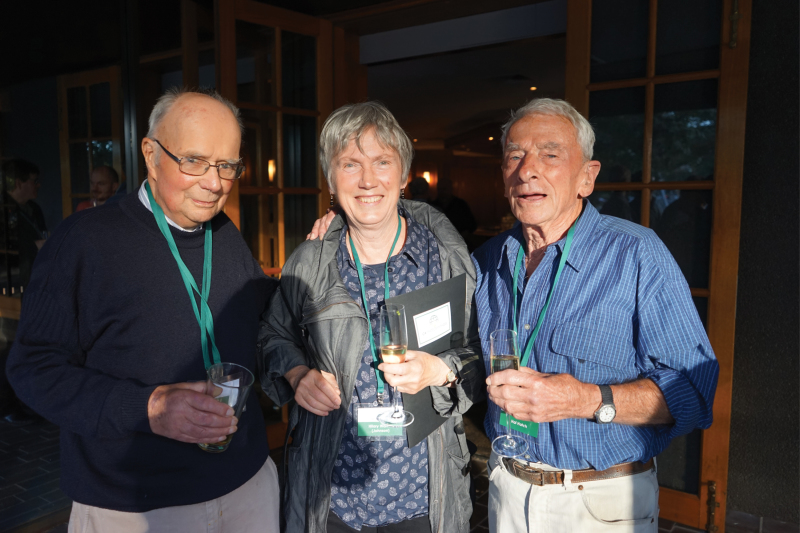

The C_4_ pathway isn’t just about biochemistry, rather it is a complex combination of biochemical and morphological specialization. Most C_4_ species are characterized by the so-called Kranz anatomy, with Rubisco located in specialized cells adjacent to the vascular tissue (bundle sheath cells) and PEP carboxylase in the mesophyll cells. It is the gas-tight nature of the bundle sheath that allows the decarboxylation of C_4_ acids in this compartment to elevate CO_2_ partial pressure around Rubisco. This inhibits its oxygenase activity allowing it to operate close to its maximal rate.

Despite this complexity, C_4_ photosynthesis is recognized as one of the most dynamic examples of convergent evolution, arising multiple times over the last 60 million years in warm semi-arid regions, with early occurrences coinciding with low atmospheric CO_2_ in the late Oligocene (Sage *et al.*, 2011; Sage, 2016). In his Darwin review, Sage (2016) outlines the evolution of the 61 independent C_4_ lineages which have resulted in more than 8000 species in grasses, sedges and eudicots and looks at the biogeography of these species.

C_4_ plants play a key role in world agriculture – crops such as maize and sorghum are major contributors to world food production in both developed and developing nations, and the C_4_ grasses sugarcane, miscanthus and switchgrass are the major plant sources of bioenergy. In comparison to C_3_ crops such as rice, C_4_ crops have higher yields and increased water and nitrogen use efficiency (Hibberd *et al.*, 2008; Langdale, 2011). The agronomic use of C_4_ species, as well as their substantial influence on terrestrial CO_2_ fixation (Still *et al.*, 2003), provides the scientific drive for understanding what has allowed the evolution of C_4_ photosynthesis to happen so many times.

This special issue follows two other recent volumes of *Journal of Experimental Biology* focused on C_4_ (‘Exploiting the engine of C_4_ photosynthesis’ – Volume 62, Issue 9, see Sage and Zhu, 2011; and ‘C_4_ and CAM photosynthesis in the new millennium’– Volume 65, Issue 13, see Sage, 2014). The papers here continue the C_4_ story and highlight the diversity of current research in the quest to get a better understanding of the C_4_ photosynthetic process and enable crop scientists to perhaps imitate the process of C_4_ evolution and turn C_3_ plants into C_4_ plants (Box 2).

**Box 2. The multidisciplinary approaches used and needed to unravel the secrets of C_4_ photosynthesis**

The C_4_ photosynthesis conference in Canberra in 2016 brought together world experts in the field ranging in discipline across biochemistry, physiology, molecular genetics and ecophysiology, and also included those involved in applied efforts to engineer C_4_ into C_3_ crops. Currently, a renewed research focus on C_3_–C_4_ intermediate species is unearthing more intermediate species and new evidence for the molecular transition from the C_3_ to the C_4_ state. The description of improved mathematical models, combined gas exchange and stable isotope tools, metabolic ^13^CO_2_ labelling kinetics and more efficient transformation systems for C_4_ plants (such as *Setaria viridis*) hold great promise for improving C_4_ photosynthesis in a crop environment.

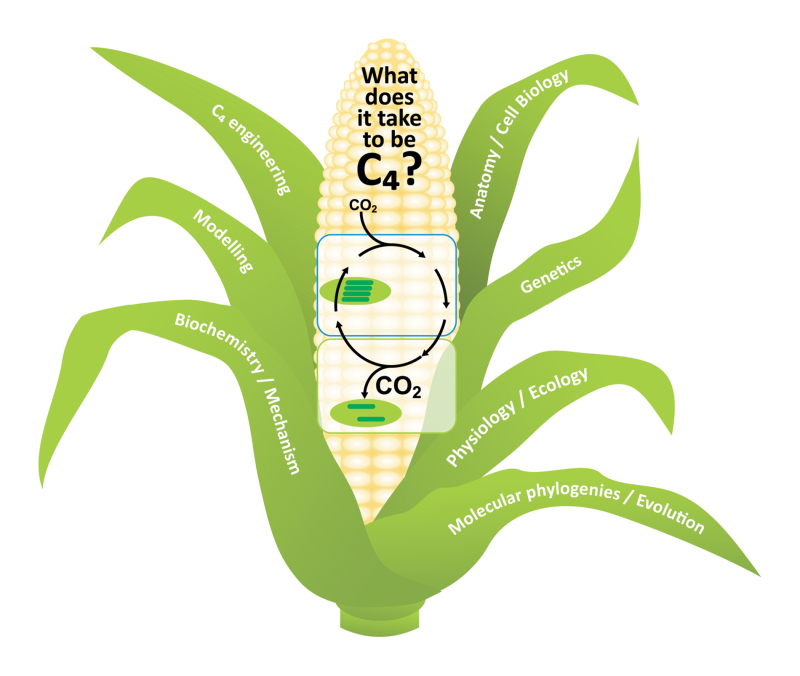

## What can we learn from genomes and gene regulation in C_4_ species?

Furbank (2016) points to a wealth of genomic and transcriptomic information now available for C_4_ leaves, and leaves of closely related C_3_ plants, which is catalysing a new generation of research into the C_4_ mechanism and the genetic architecture underpinning it. A transcriptomics/genomics approach and a review of gene expression across multiple lineages of C_4_ plants (Aubry *et al.*, 2014; Williams *et al.*, 2016; Reeves *et al.*, 2017) have led to the conclusion that regulation of gene expression at multiple levels (including transcriptional control by promoter regions, as demonstrated by Gowik *et al.*, 2017) is required for C_4_ photosynthesis to function. It appears that posttranscriptional control may also be important (Fankhauser and Aubry, 2017) and that many of the mechanisms for regulation of C_4_ gene expression are indeed present in C_3_ plants and recruited to a C_4_ function (Reeves *et al.*, 2017). Denton *et al.* (2017) remind us, though, that caution must be used in interpreting gene expression data, particularly cell- or tissue-specific data, which may include biases due to RNA preparation methods. The utility of comparative genomics in this field is shown by Huang *et al.* (2017), who have developed a cross-species genome scanning approach to identify genes under positive selection in C_4_ evolution which is independent from knowledge of the biochemical pathways involved (see also the Insight article in this issue by Christin, 2017). It is interesting that molecular genetics, genomics and transcriptomics are now commonly being used in a biochemical and evolutionary research perspective as affordable approaches to answer questions in C_4_ photosynthesis research, rather than operating in isolation as stand-alone fields (Box 2).

## New insights from phylogeny and C_3_–C_4_ intermediate species

Since their discovery, C_3_–C_4_ intermediate species have been hypothesized to be evolutionary intermediates on the path to or from C_4_ photosynthesis (Peisker, 1986; Monson and Moore, 1989; Sage *et al.*, 2012; Heckmann *et al.*, 2013). Biochemical and molecular studies have elucidated the various types of photosynthetic intermediacy, which range from the simpler C_2_ mode involving the glycine or photorespiratory shuttle with rudimentary bundle sheath to a C_4_-like pathway with well-developed Kranz anatomy and functional C_4_ pathway (Sage *et al.*, 2012). Physiological studies have revealed a clear lowering of the CO_2_ compensation point (CO_2_ partial pressure where there is no net CO_2_ exchange) for all types of C_3_–C_4_ intermediates, but advantages related to improved water and nitrogen use efficiency are only expressed in intermediate plants possessing a degree of C_4_ acid fixation (Vogan *et al.*, 2011; Pinto *et al.*, 2016). More recent studies, including those represented in this issue, have focused on documenting the phylogenetic diversity of C_3_–C_4_ taxa and elucidating the molecular elements underscoring the evolutionary, and in rare cases, the developmental, transitions from C_3_ to C_4_ (Gowik *et al.*, 2011). The prized goal has been the mining of C_3_–C_4_ species to identify anatomical, biochemical and molecular features that underlie C_4_ evolution.

In this issue, both Voznesenskaya *et al.* (2017) and Schüßler *et al.* (2017) report on phylogenetic searches for C_3_–C_4_ species. Voznesenskaya *et al.* (2017) demonstrate that the family Portulacaceae has a C_3_–C_4_ Cryptopetala clade and a diverse C_4_ Pilosa clade, while Schüßler *et al*. (2016) resolve the C_3_–C_4_ intermediate and C_4_ lineages in the Salsoleae family (Chenopodiaceae). Lauterbach *et al.* (2017) go a step further and combine physiological, anatomical and transcriptomic approaches to elucidate the molecular transition from the C_3_ to the C_4_ state in the leaves of *Salsola soda* (Chenopodiaceae). Kümpers *et al.* (2017) use leaf maturation in C_3_ and C_4_ *Flaveria* species to identify transcription factors. Schlüter *et al.* (2017) draw our attention to limitations connected to N metabolism and vein density that may have constrained the evolutionary transition of two *Moricandia* species (Brassicaceae) from C_3_–C_4_ into the C_4_ pathway. Regardless of phylogenetic constraints, Lundgren and Christin (2017) demonstrate that the evolution of the C_3_–C_4_ pathway brings intermediate species into C_4_-like environments facilitating C_4_ evolution.

## New technologies and mathematical models elucidate the physiology and biochemistry of C_4_ photosynthesis

Early discoveries of C_4_ photosynthesis made use of new physiological techniques such as gas exchange measurements. This led to the development of distinguishing gas exchange features of C_4_ CO_2_ assimilation rates. We now have a good understanding of how C_4_ photosynthesis responds to environmental variables such as light, temperature and CO_2_ (Long, 1999). The first biochemical model of C_4_ photosynthetic gas exchange correctly predicted its CO_2_ concentrating function, with first estimates of bundle sheath CO_2_ partial pressures, although we still don’t know what they actually are (Berry and Farquhar, 1978). These functional models of C_4_ which allow the link between leaf biochemistry and gas exchange have become essential tools (von Caemmerer and Furbank, 1999; von Caemmerer, 2000). Bellasio (2017) has combined these models to generate a general stoichiometric model for C_3_, C_2_, C_2_+C_4_, and C_4_ photosynthesis in which energetics, metabolite traffic and the different decarboxylating enzymes are explicitly included. The model comes with an Excel spreadsheet inviting the community to have a go at redesigning C_4_ photosynthesis. The usefulness of a sound mathematical framework is also highlighted in the opinion paper by Li *et al.* (2017), who use these models (following Heckmann *et al.*, 2013) to outline how to combine genetic and evolutionary engineering to establish C_4_ metabolism in C_3_ plants.

Most C_4_ species are characterized by Kranz anatomy, but there are a small number, such as *Bienertia cycloptera*, that perform C_4_ photosynthesis within individual mesophyll cells (Voznesenskaya *et al.*, 2001; King *et al.*, 2012). By modelling the processes of diffusion, capture and release of CO_2_ and oxygen inside a typical *Bienertia* mesophyll cell, Jurić *et al.* (2017) show that a spatial separation as low as 10 μm between the primary and the secondary carboxylases can provide enough diffusive resistance to sustain an efficient C_4_ pathway, demonstrating that single-cell C_4_ photosynthesis is a viable option. CO_2_ diffusion during C_4_ photosynthesis also remains an important issue in those species with Kranz anatomy. Theories developed for the interpretation of stable isotope discrimination during C_4_ photosynthesis (Farquhar, 1983; Gillon and Yakir, 2000; Barbour *et al.*, 2016) allow us to probe the interconnectivity of C_3_ and C_4_ cycle activity and CO_2_ diffusion properties into mesophyll cells. For example, ^13^CO_2_ isotope discrimination can be used to quantify bundle sheath leakiness (the ratio of CO_2_ leak rate out of the bundle sheath over the rate of CO_2_ supply) and C^18^OO discrimination allows quantification of CO_2_ diffusion from intercellular airspace to the mesophyll cytosol in relation to carbonic anhydrase activity there. Recent technical advances have greatly facilitated the measurements of isotope discrimination concurrently with gas exchange (Gong *et al.*, 2017; Osborn *et al.*, 2017). Gong *et al.* (2017) documented dynamic variation in bundle sheath leakiness of a perennial C_4_ grass with short-term variation in atmospheric CO_2_ concentration. Osborne *et al.* (2017) generated transgenic *Setaria viridis* plants with reduced carbonic anhydrase activity and used measurements of C^18^OO discrimination to show that carbonic anhydrase and mesophyll conductance are both limiting factors affecting CO_2_ assimilation rates at low CO_2_ partial pressures.

The techniques of the ^14^C pulse chase which were used by Hatch and Slack to unravel the mysteries of C_4_ photosynthesis have been replaced by mass spectrometric measurements of ^13^CO_2_ labelling kinetics, which provide a wealth of information compared to past experiments. This technique was used for the first time by Arrivault *et al.* (2017) in maize to establish pool sizes and gradients of metabolites using cell type fractionation. It will provide a welcome tool for establishing C_4_ metabolism in C_3_ species.

Today, major C_4_ crops are grown in dense stands where most leaves are shaded compared to their wild progenitors. Pignon *et al.* (2017) show that leaves of two highly productive C_4_ crops lose photosynthetic efficiency in low light as they become shaded by new leaves, costing the crop up to 10% of its yield potential. The ancestors of maize and miscanthus appear to have existed in very open habitats, where water and nutrient deficiencies would have limited leaf area. There may therefore have been little evolutionary pressure for maintenance of photosynthetic efficiency in shade conditions. Improving C_4_ photosynthesis in a crop environment may be an important next step for increasing genetic yield potential in some of these most important crops (Long *et al.*, 2015; von Caemmerer and Furbank, 2016).

## Future perspectives

Where will C_4_ research go next? In 50 years we have seen the expansion of the field from the examination of a rudimentary biochemical pathway in just a few species to the construction of complex evolutionary models and assembly of massive genomic and transcriptomic data sets from a large range of both crop and wild C_4_ species, as well as multiple efforts to engineer C_4_ traits into C_3_ crops and model species. As gene and transcript sequencing costs plummet with third-generation technologies, what will be the new technological driver of C_4_ research?

From a biochemical and modelling perspective, the confounding nature of the two-compartment C_4_ system for ‘grind and find’ extraction of metabolites, transcripts and proteins has been a challenge. Recently, however, high resolution MALDI imaging mass spectrometry was used to examine the lipid composition of thylakoids of mesophyll and bundle sheath cells of maize (Dueñas *et al.*, 2016). With appropriate rapid kill and cryopreservation, this technique may hold promise for measuring metabolites during photosynthesis in mesophyll and bundle sheath compartments more accurately.

Gene discovery through genomics approaches reveals gene candidates and evidence for the importance of certain genes in evolution or for plant performance, but these must be experimentally validated. Commonly, for C_3_ dicots, this is done in model systems like Arabidopsis or tobacco by gene inactivation or overexpression, but only recently have grass transformation systems become sufficiently routine for researchers to approach these experiments in their laboratories. Alternatives such as producing large panels of mutants by non-targeted mutagenic approaches or by crossing genetic material to develop near-isogenic lines with and without genetic polymorphisms is outside the scope of most small research laboratories. Future development of new and more efficient transformation systems for a range of C_4_ plants and the development of genetic stocks which can be ordered routinely for knockout lines and backcrossed mutants, sequenced populations and recombinant in-bred lines would see a rapid development in C_4_ research similar to that seen when Arabidopsis genetic resources became widely available.

In the field of C_4_ engineering, synthetic biology has the potential to impact hugely on both basic and basic/strategic engineering approaches (Schwander *et al.*, 2016). In the case of the C_4_ rice project, the ability to make multiple gene constructs simplifies cloning strategies (Simkin *et al.*, 2015). Similarly, if CRISPR/Cas9 technology is combined with high-efficiency C_4_ grass transformation systems, production of allele mimics of potentially important genes occurring in nature and engineering of novel enzyme properties in C_4_ plants would advance rapidly.

There is a vast array of information and new technology now at our fingertips. Nevertheless, we must still marvel at the achievements of researchers 50 years ago in assembling a completely new photosynthetic pathway from a collection of radiolabelling experiments and enzyme assays, and the rapidity with which these researchers brought C_4_ anatomical and biochemical data together to underpin the knowledge of the C_4_ mechanism we have today.
